# Faecal levels of calprotectin in systemic sclerosis are stable over time and are higher compared to primary Sjögren’s syndrome and rheumatoid arthritis

**DOI:** 10.1186/ar4475

**Published:** 2014-02-06

**Authors:** Kristofer Andréasson, Tore Saxne, Agneta Scheja, Izabela Bartosik, Thomas Mandl, Roger Hesselstrand

**Affiliations:** 1 Department of Clinical Sciences Lund, Section of Rheumatology, Lund University, Lund, Sweden; 2 Department of Clinical Sciences Malmö, Section of Rheumatology, Lund University, Lund, Sweden

## Abstract

**Introduction:**

Faecal calprotectin (FC) has been proposed to be a biomarker of gastrointestinal (GI) disease in systemic sclerosis (SSc). The purpose of this study was to extend cross-sectional observations and prospectively assess the variability of FC over time in SSc patients. We also aimed to examine FC in relation to immunosuppressive therapy. Finally we wanted to analyse FC in other rheumatic diseases to evaluate the specificity of FC for SSc GI disease.

**Methods:**

FC was measured in consecutive patients with SSc, primary Sjögren’s syndrome (pSS), rheumatoid arthritis (RA) and in healthy hospital workers. The intraindividual variability of FC in SSc was assessed with intra class correlation (ICC) and κ statistics. Associations between FC and objective markers of GI disease and immunosuppressive medication were investigated.

**Results:**

FC was associated with micronutrient deficiency and GI pathology as assessed by cineradiography confirming our previous results. FC showed only a limited intra-individual variation in SSc, ICC = 0.69 (95% confidence interval, CI: 0.57-0.78) and κ = 0.64 (95% CI: 0.56-0.73). Generalised immunosuppression did not have any significant impact on FC. FC was significantly higher in SSc patients compared to patients with pSS or RA as well as compared to healthy subjects.

**Conclusions:**

FC is a promising non-invasive biomarker for GI disease in SSc. In view of stable levels over time, FC could be a useful marker when novel, more specific drugs targeting the GI tract in SSc will be introduced.

## Introduction

Faecal calprotectin (FC) is a validated non-invasive biomarker in inflammatory bowel disease (IBD) [1,2]. Recently, we presented FC as a possible biomarker of gastrointestinal (GI) disease in systemic sclerosis (SSc). In that cross-sectional study, we showed significant correlations between FC and objective markers of SSc GI disease, that is, cineradiography and micronutrient deficiency [3].

GI manifestations of SSc are common and a major concern in this disease [4]. Recent studies have suggested that 90% or more of SSc patients have GI complications and GI involvement is a recognised cause of SSc-related death [5-9].

Symptomatic treatment of several GI manifestations of SSc is available, but current treatment regimens are insufficient [10,11]. One problem in developing or identifying new drugs in this field is the lack of both objective and feasible biomarkers to monitor SSc GI disease over time [12].

Calprotectin, also known as s100A8/A9, MRP8/14 or calgranulin A/B, is a heterodimer composed of two small proteins. It constitutes a major (40% to 60%) part of the cytosolic proteins in neutrophils and is also present in other myeloid cells. Calprotectin is released from leukocytes both upon activation and cell death and levels of FC correlate to leukocyte influx in the GI lumen [13,14]. The calprotectin heterodimer is a stable complex inert to degradation in stool even when kept at room temperature for several days [15]. Highly increased levels of FC are typical in active IBD, but increased levels have been reported in some other inflammatory pathologies in both the upper and lower GI tract including gastric ulcer, GI cancer and bacterial gastroenteritis, but notably not small intestinal bacterial overgrowth [16,17]. Repeated testing of FC in IBD has proven that the biomarker co-varies with disease activity [18]. In the field of gastroenterology, FC is today a recognised clinical tool to investigate patients with unspecific bowel symptoms, to monitor IBD over time and to evaluate response to different treatment regimens [1]. In IBD, immunosuppressive therapy is often successful and accompanied by a decrease in FC. In SSc however, immunosuppressive therapy has not been shown to modify the course of the GI disease.

The primary aim of the present study was to extend our cross-sectional study and prospectively assess the variability over time of FC in SSc patients. A second aim was to monitor FC in relation to conventional immunosuppressive therapy in SSc. A third aim was to examine FC in other rheumatic diseases, that is, rheumatoid arthritis (RA) and primary Sjögren’s syndrome (pSS) in order to evaluate the specificity of pathological FC testing for SSc GI disease in a rheumatological setting. For comparison, FC was also assessed in healthy hospital workers.

## Methods

### Patients

This study was conducted at the Rheumatology Clinic, Skåne University Hospital, Sweden, which is a referral centre for SSc [19]. All patients admitted to in-hospital care for evaluation of SSc between September 2008 and February 2013, fulfilling the 1980 American College of Rheumatology (ACR) criteria for SSc were invited to deliver a stool sample for analysis of FC upon each visit to the clinic. Patients who had FC measured at least twice during this time period were included in the study.

Consecutive patients with pSS according to the American-European Consensus Classification Criteria, [20] visiting the outpatient Rheumatology Clinic, Skåne University Hospital Malmö, between June and December 2012, were included in the study and delivered a stool sample after their clinical visit.

Consecutive patients with RA, positive for rheumatoid factor or anti-cyclic citrullinated peptide antibodies, awaiting either initiation of anti-TNF-α-therapy or orthopaedic surgery at the Rheumatology Clinic, Skåne University Hospital Lund, were invited to participate in this study as a second reference group to the SSc cohort.

Patients with a history of IBD, GI malignancy, diverticulitis or alcohol abuse were excluded.

Finally, healthy hospital workers, not currently treated with non-steroidal anti-inflammatory drugs (NSAIDs) or proton pump inhibitors (PPI) were asked to participate in this study.

### Clinical assessment

Sex and age at time of stool delivery were recorded for all subjects.

From all SSc patients, the following data were collected: disease duration (defined as years since debut of first non-Raynaud’s manifestation), autoantibody profile and results from cineradiography. This examination has been proposed to be a core set variable in the clinical evaluation of the GI tract in SSc [21]. Cineradiography reports were independently categorised by RH and AS, who were blinded with regard to patient data. Cineradiographies were analysed in reference to motility. Investigations showing signs of disturbed motility were regarded as pathological, and investigations with severe dysfunction of the oesophagus, that is, aperistalsis, were classified as severely pathological. Investigations with no signs of disturbed motility were regarded as normal. Investigations were regarded as inconclusive if RH and AS did not both agree that a cineradiography was normal or pathological. When RH and AS disagreed on the severity of pathology, the evaluation of the senior physician was chosen.

As we have previously studied FC in relation to the modified Rodnan skin score, autoantibody status and disease subtype without any associations identified, these issues were not further investigated [3].

All pSS patients were categorised according to the European League against Rheumatism (EULAR) Sjögren’s Disease Activity Index (ESSDAI) and the Disease Activity Score 28 based on C-reactive protein (CRP) and consequently, and three variables (DAS28-CRP (3)) was calculated for all RA patients [22,23].

### Laboratory examinations

FC was measured in stool samples with a commercially available ELISA using a monoclonal antibody (Bühlmann Laboratories, Schönenbuch, Switzerland). The lower detection limit was 30 μg/g. Analyses were done at the Department of Immunology, Skåne University Hospital, Lund.

Different cut-off levels of FC indicating organic disease have been suggested. Two cut-off levels were used in this study. Based on the majority of reports where the current ELISA has been used, FC levels above 50 μg/g were considered pathological [1,16,24]. Based on current recommendations in Swedish practice, as well as international suggestions/experience, we also used a second cut-off level of 200 μg/g to indicate substantial FC elevation [25,26].

All SSc patients were subject to analysis of the following micronutrient deficiencies by laboratory examination of serum or plasma: iron, vitamin B_12_, zinc and folic acid. Subjects with pathological testing according to local laboratory standards were regarded as micronutrient deficient. Subjects with an iron/total iron-binding capacity ratio <0.16 were considered to be iron deficient [27]. Patients with pathological testing for more than one of the aforementioned micronutrients were categorised as having multiple deficiencies.

### Ethics

The study was approved by the Lund University Ethics Committee, Lund, Sweden. All patients and healthy volunteers gave their written permission according to the Declaration of Helsinki to be included in the study.

### Statistics

As previously described, the distribution of the log-transformed FC values allowed parametric statistics [3]. Student’s t-test, 1-way analysis of variance and Pearson correlation coefficient could thus be used and comparative data are thus presented in geometric means. Paired t-test was used when comparing repeated FC measurements in the same patients. Consistency was measured with intraclass correlation (ICC) and kappa statistics (κ). FC values <30 μg/g were approximated as 20 μg/g in all analyses. When calculating the ICC, separate data are presented for which FC values <30 μg/g are excluded. Median and interquartile range (IQR) was used for descriptive data. A two-sided *P*-value of 0.05 was considered significant and 95% confidence intervals (CI) given when possible. Because of the interdependence between different variables analysed in this study, we chose not to use Bonferroni correction. All analyses were made using IBM SPSS Statistics version 20 (IBM Corporation, Somers, NY, USA).

## Results

In total, 93 SSc patients had FC measured at least twice during the study period. Of these patients, FC had been measured three times in 57 patients and at least four times in 27 patients. The main characteristics of the patients involved in this study are presented in Tables 1 and 2.

**Table 1:** Characteristics of study participants

	**Age, years median (IQR)**	**Female sex, number (%)**	**Faecal calprotectin, μg/g, median (IQR)**	**ESSDAI/DAS28-CRP(3), median (IQR)**
**Systemic sclerosis (n = 93)**	62 (51 to 70)	77 (83)	110 (42 to 210)	NA
**Primary Sjögren’s syndrome (n = 44)**	61 (55 to 68)	43 (98)	41 (<30 to 118)	7 (1 to 10)
**Rheumatoid arthritis (n = 24)**	66 (55 to 69)	20 (83)	43 (<30 to 93)	3.1 (2.1 to 3.9)
**Healthy controls (n = 21)**	56 (47 to 58)	17 (81)	<30 (<30 to 56)	NA

DAS28-CRP(3), Disease Activity Score 28 based on CRP and 3 variables; ESSDAI, EULAR Sjögren’s Disease Activity Index; IQR, interquartile range; NA, not applicable.

**Table 2:** Characteristics of SSc patients

**Characteristic**	**Number (%)**
Diffuse: limited skin involvement	20:73 (22:78)
Anti-centromere antibodies	30 (32)
Anti-topoisomerase antibodies	22 (24)
Anti-RNA polymerase III antibodies	3 (3)
Pathological cineradiography	70 (75)
Immunosuppressive therapy	36 (39)
	**Median (IQR)**
Disease duration (years)	7 (2 to 13)

IQR: interquartile range.

### FC correlates with objective variables of GI disease in SSc

**Figure 1:**
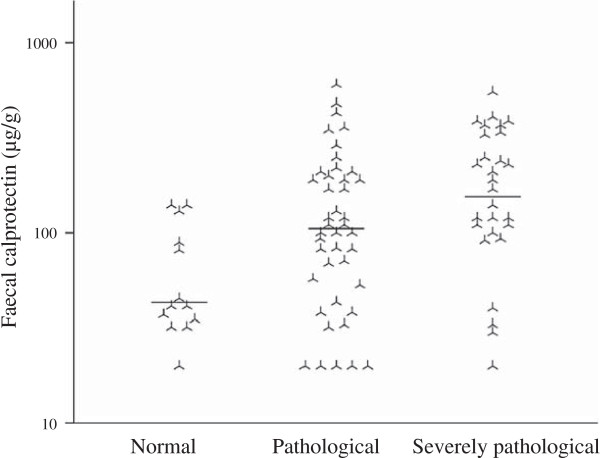
**In systemic sclerosis, faecal calprotectin is associated with pathological cineradiograhy.** Individual and median faecal calprotectin levels in systemic sclerosis patients with different degrees of pathological cineradiographies. 1-way analysis of variance, *P* = 0.007. Please note the logarithmic scale on the y-axis.

**Figure 2:**
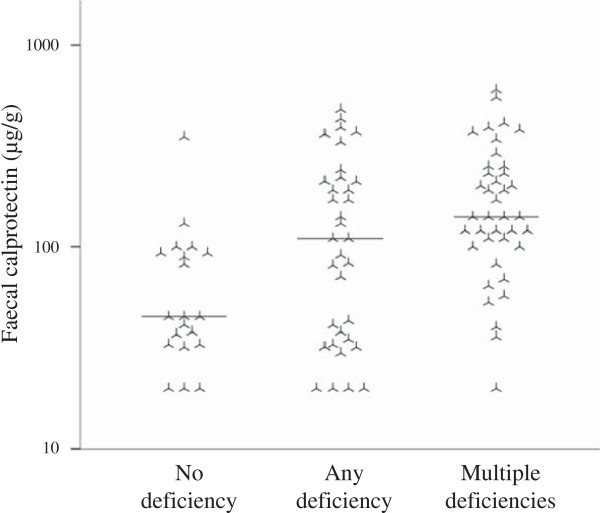
**In systemic sclerosis, faecal calprotectin is associated with micronutrient deficiency.** Individual and median faecal calprotectin levels in systemic sclerosis patients with different degrees of micronutrient deficiency. 1-way analysis of variance, *P* = 0.005. Please note the logarithmic scale on the y-axis. Patients were subject to analysis of the following micronutrient deficiencies by laboratory examination of serum or plasma: iron, vitamin B_12_, zinc and folic acid. Subjects with pathological testing according to local laboratory standards were regarded as micronutrient deficient. Patients with pathological testing for more than one of the aforementioned micronutrients were categorised as having multiple deficiencies.

Eighty-three subjects had conclusive cineradiographies. Of these, patients with a pathological investigation had higher levels of FC (geometric mean 119 versus 53 μg/g, *P* = 0.010), Figure 1. In eight cases RH and AS did not agree if the investigation was pathological or not, and in another five cases RH and AS did not agree on the severity of a pathological examination. Out of thirteen patients with normal investigations, five patients had pathological FC levels. Among the remaining 70 patients with pathological cineradiographies, 56 patients had pathological FC levels. Patients with micronutrient deficiency had higher FC values compared to micronutrient sufficient patients (geometric mean 118 versus 50, *P* = 0.001), Figure 2.

### FC shows a low degree of intra-individual variation over time in SSc

**Figure 3:**
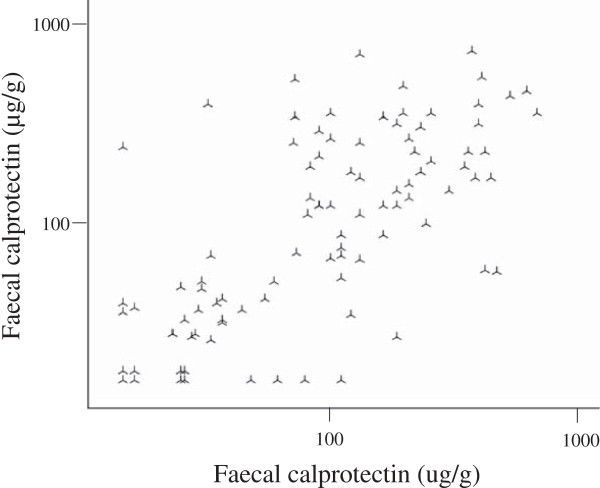
**Repeated testing of faecal calprotectin in systemic sclerosis.** Faecal calprotectin at time point 2 as a function of faecal calprotectin at time point 1 in 93 SSc patients. ICC = 0.69 (95% CI: 0.57 to 0.78). Please note the logarithmic scale on the x- and y-axis. CI, confidence interval; ICC, intra class correlation.

The median (IQR) time span between the first (FC1) and the second (FC2) stool delivery was 369 (318 to 725) days. We saw only limited variation in FC over time (Figure 3) and the reliability of FC to classify patients as having normal or pathological FC levels was substantial, κ = 0.64 (95% CI: 0.56 to 0.73, n = 93) [28].

The consistency between FC1 and FC2 was good both when all patients were included, ICC = 0.69 (95% CI: 0.57 to 0.78), and when cases <30 μg/g were excluded, ICC = 0.62 (95% CI: 0.46 to 0.74) [29]. We saw no tendency of FC to increase or decrease with time as FC1 and FC2 levels were similar (geometric mean 130 versus 125 μg/g, *P* = 0.625). This was also true for patients with disease duration less than 400 days (n = 15) at time point 1, where no increase in FC could be seen over a median (IQR) time period of 358 (229 to 385) days (geometric mean 83 versus 96 μg/g, *P* = 0.437). Notably, however, 11/15 of these early patients had pathological FC levels already at baseline.

The consistency and reliability of FC was not superior in patients with a short timespan compared to patients with a long timespan between FC1 and FC2 (data not shown).

A separate analysis of patients who had FC measured at least three times further indicated that patients with normal, pathological or substantially elevated FC levels were also likely to show a similar FC result upon repeated testing (Table 3).

**Table 3:** Faecal calprotectin is stable over time in systemic sclerosis

	**Typical FC level**			
**Variability of FC over time**	**Normal**	**Elevated**	**Substantially elevated**	**All patients, n (%)**
	**FC < 50, n**	**FC = 50-200, n**	**FC >200, n**	
Cohort A, n = 57				
Solid^a^	6	7	6	19 (33)
Stable^b^	3	18	12	33 (58)
Inconclusive^c^	0	4	1	5 (9)
All patients	9	29	19	57 (100)
Cohort B, n = 28				
Solid^a^	4	1	2	7 (25)
Stable^b^	1	7	6	14 (50)
Inconclusive^c^	0	4	3	7 (25)
All patients	5	12	11	28 (100)

^a^All FC measures fall into the same category; ^b^all FC measures fall into the same category except 1 that is 1 step lower/higher; ^c^the FC measures show higher variability than above. Stool samples were delivered for analysis of FC at three (Cohort A) or four (Cohort B) time points and FC was categorised as solid^a^, stable^b^ or inconclusive^c^. FC: faecal calprotectin measured in μg/g.

### Initiation or cessation of immunosuppression is not associated with a change in FC

During the study, eight patients had FC measured both before and after initiation of immunosuppressive therapy. Median (IQR) disease duration at FC1 was 295 (156 to 575) days and the second FC was delivered 339 (190 to 434) days later. Immunosuppression was initiated because of interstitial lung disease, extensive skin fibrosis, myositis, arthritis, serositis or any combination of these manifestations, but not because of gastrointestinal disease. Four patients started immunosuppression with azathioprine, three with mycophenolate mofetil and one with cyclophosphamide.

FC was not different before compared to after initiation of immunosuppressive therapy (geometric mean 41 versus 71 μg/g, *P* = 0.243).

During the study, 15 patients delivered FC both before and after cessation of immunosuppressive therapy. The majority of the patients had been prescribed immunosuppressants because of interstitial lung disease and no one because of gastrointestinal disease. A majority of the patients (9/15) had been prescribed mycophenolate mofetil, four had been prescribed azathioprine and two cyclophosphamide. FC was not different before compared to after cessation of immunosuppression (geometric mean 112 versus 130 μg/g, *P* = 0.380).

Twenty-six SSc subjects were taking glucocorticoids at FC1; no relationship between FC and glucocorticoid intake was noted (r = 0.14, *P* = 0.182).

### FC is higher in SSc compared to pSS, RA and healthy controls

**Figure 4:**
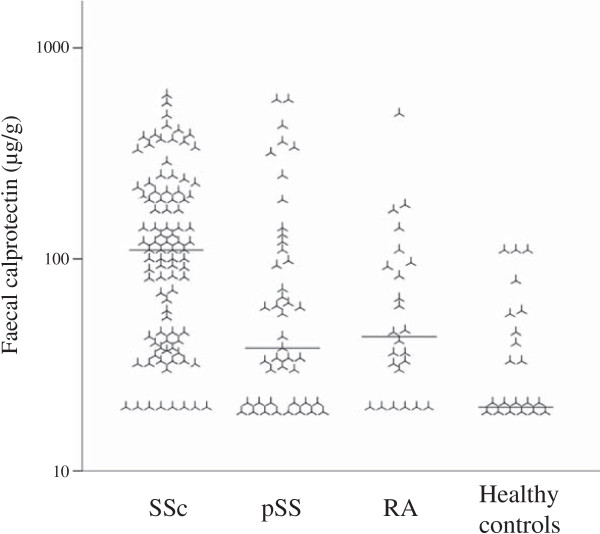
**Faecal calprotectin in different patient groups.** Individual and median levels of faecal calprotectin in patients with systemic sclerosis (SSc) n = 93, primary Sjögren’s syndrome (pSS) n = 44, rheumatoid arthritis (RA) n = 24 and healthy controls n = 21. Please note the logarithmic scale on the y-axis.

FC was measured in patients with pSS (n = 44) and RA (n = 24), and healthy controls (n = 21) (Table 1 and Figure 4). FC was significantly higher in SSc compared to any of the three other groups (*P* < 0.005 for each comparison).

Of note, patients with pSS had significantly higher FC values than healthy controls (geometric mean 57 versus 34 μg/g, *P* = 0.019). Furthermore, FC correlated with ESSDAI in these patients (r = 0.52, *P* < 0.001). Subgroup analysis of the different ESSDAI-components did not indicate any specific domain score of the ESSDAI to be solely responsible for this correlation. There was a weak correlation between FC and erythrocyte sedimentation rate (r = 0.31, *P* = 0.048), but no correlation between FC and immunoglobulin G (IgG) (r = 0.04, *P* = 0.785). There was no significant difference in FC between users and non-users of NSAIDs or PPI (geometric mean 43 versus 68 μg/g, *P* = 0.108 and 69 versus 52 μg/g, P = 0.399, respectively). Finally, among the pSS patients, FC did not correlate with glucocorticoid intake (r = 0.19, *P* = 0.211).

In RA, FC was not significantly higher than in healthy controls (geometric mean 52 versus 34 μg/g, *P* = 0.069) and did not correlate with disease activity measured as DAS28-CRP(3) (r = 0.06, *P* = 0.788). There was a significant correlation between FC and glucocorticoid intake (r = 0.66, *P* < 0.001), in contrast to patients with pSS or SSc. RA subjects taking NSAIDs or PPI did not have significantly higher FC levels compared to non-users (geometric mean 55 versus 46 μg/g, *P* = 0.666 and 62 versus 45 μg/g, *P* = 0.388 respectively).

## Discussion

In the present study we have assessed repeated testing of FC as a biomarker for SSc GI disease in 93 patients showing low variability over time. In order to be of clinical value, a biomarker should be objective, feasible, show construct and face validity, be stable over time but also sensitive to change if disease severity or activity is altered [30]. FC is an objective biomarker that because of its wide spread use in gastroenterology is readily available in the clinic.

In this study we confirm our earlier results showing significant correlations between FC and objective signs of SSc GI disease as determined by cineradiography and testing for micronutrient deficiency. These data imply construct validity of FC for SSc GI disease.

FC is measured in stool samples originating from the GI tract which is the very organ system implicated in SSc GI disease suggesting face validity.

The intra-individual variability of FC has been studied in a cohort of patients with quiescent Crohn’s disease with a similar distribution of FC as our SSc subjects [31]. These patients were tested on consecutive days and the ICC and κ statistic were thus projected mainly to reflect methodological rather than biological variation. The ICC and κ statistic in that study (0.84 and 0.648) are similar to our results, 0.62 and 0.64, respectively. These results indicate not only substantial consistency of repeated FC testing in SSc, but also that the intra-individual variation of FC in SSc is low even when testing is separated by longer time periods.

FC has been shown to be a feasible test in IBD [32]. All patients in the present study were examined at least twice, 57 patients three times, and 28 patients four times. The patients were motivated to deliver stool samples and our experience during this study is that multiple testing of FC in this in-hospital setting is as feasible in SSc, as has been shown in IBD.

To date, no drug has been shown to modulate the basic mechanisms behind GI manifestations of SSc [7,10]. The poor response of SSc GI disease to immunosuppression was reflected by FC, as we could not identify any association between FC and initiation or cessation of immunosuppressive therapy. Both the behaviour of FC and the clinical GI picture are thus different in SSc compared to IBD in relation to immunosuppressants. This is a crucial requisite if FC is to function as a biomarker in SSc.

While we show that FC is stable over time, we have yet to show if FC is sensitive to change. Our findings may suggest that any drug affecting FC levels in SSc may actually modulate the GI tract in a unique way and could thus have potential as a drug that suppresses SSc GI disease.

Calprotectin levels in serum and joint fluid in RA and osteoarthritis have attracted considerable interest, but less is known about faecal levels of calprotectin in rheumatic diseases [33,34]. An initial report on the methodology of FC testing in 1992 indicated increased levels of FC in RA patients [15]. FC has also been studied in patients with ankylosing spondylitis, of whom, interestingly, a majority presented pathological FC testing [35,36].

Our data indicate that pathological FC testing is more common in SSc compared to pSS and RA. Still, FC levels were higher in pSS compared to healthy controls, and FC is not specific for SSc in an unselected rheumatological setting. In RA, we identified a significant, dose dependent association between FC and glucocorticoid usage. Associations between FC and NSAID usage have been reported, but to our knowledge not studied in patients with RA or pSS before. Similar to our previous report in SSc, we did not identify any association between FC and NSAIDs that can explain our findings in pSS and RA [3,35,37]. These findings need further investigation and question the generalisability of proposed cut-off levels of FC for patients with chronic diseases under different medical therapies.

pSS is a systemic autoimmune disease characterised by inflammation of the exocrine glands which also can involve the GI tract. The pathogenesis behind GI disease in pSS has been suggested to be quite different compared to SSc. While autonomic nervous dysfunction [38] and GI dysmotility [39] are common in both diseases no inflammatory component similar to SSc has been described [40]. Our findings support the notion that the inflammatory component of SSc GI pathology is different compared to pSS.

Several organ manifestations of SSc change in both intensity and characteristics over time, most notably scleroderma of the skin, lung fibrosis and pulmonary arterial hypertension. We also identified pathological levels of FC in patients with early SSc. GI manifestations of SSc are reported to be common in both early and late disease. Our findings support this notion but do indicate that a possible inflammatory component could also be prevalent in late disease [41]. It can be speculated that pathological FC testing in early SSc may reflect the debut of GI involvement and thus predict later clinical manifestations of SSc GI disease. This remains to be studied further.

Some weaknesses of this study deserve to be mentioned. Several FC ELISAs are now available on the market. While little variation is seen within different laboratories, substantial variation has been documented between different laboratories in general and between different ELISAs in particular [42]. The FC measurements in this study were all done at the same laboratory using a monoclonal antibody in an ELISA that recently was shown to be superior, both in terms of specificity and sensitivity, in correctly identifying GI pathology, compared to other ELISAs [43].

The time period between the FC1 and FC2 was not standardised. This is in contrast to a recent study of FC in IBD, and warrants caution when interpreting our data [31]. Furthermore we cannot rule out the possibility of a certain selection bias since only SSc patients subject to repeated in-hospital care were recruited to the study. Finally, SSc is a heterogeneous disease. Several GI manifestations can be included in the term ‘SSc GI disease’. We cannot rule out the possibility that only a limited number of GI manifestations contribute to FC elevation in SSc. Accordingly, we do not know the origin of FC in SSc. Increased levels of FC have been described in diseases affecting both the upper and lower GI tract [16]. Autopsy and biopsy studies have shown deposition of inflammatory cells, including monocytes and neutrophils, in many segments of the GI tract in SSc [40,44]. It can be hypothesised that FC originates from such infiltrates. However, further studies are needed to investigate the exact pathological mechanisms behind increased excretion of FC in SSc.

## Conclusions

FC is a non-invasive biomarker for GI disease in SSc with only limited variation upon repeated testing. FC is higher in SSc compared to RA, pSS and healthy controls. Even though further studies are needed to determine the exact mechanisms behind pathological FC testing in SSc, FC is a promising tool for assessing novel drugs targeting the GI tract in SSc.

## Abbreviations

CI: confidence interval; CRP: C-reactive protein; DAS28-CRP(3): Disease Activity Score 28 based on CRP and 3 variables; ELISA: enzyme-linked immunosorbent assay; ESSDAI: EULAR Disease Activity Index; FC: faecal calprotectin; GI: gastrointestinal; IBD: inflammatory bowel disease; ICC: intra class correlation; IQR: interquartile range; PPI: proton pump inhibitor; pSS: primary Sjögren’s syndrome; RA: rheumatoid arthritis; SSc: systemic sclerosis; TNF-α: tumor necrosis factor-alpha.

## Competing interests

The authors declare that they have no competing interests.

## Authors’ contributions

Conception and design: KA, TS, TM, RH. Acquisition of data: all authors. Analysis and interpretation of data: all authors. Drafting of article: KA Reviewing for intellectual content: TS, TM, IB, AS, RH. All authors read and approved the final manuscript.
